# Impaired mucosal IgA response in patients with severe COVID-19

**DOI:** 10.1080/22221751.2024.2401940

**Published:** 2024-10-02

**Authors:** Melyssa Yaugel-Novoa, Blandine Noailly, Fabienne Jospin, Andrés Pizzorno, Aurélien Traversier, Bruno Pozzetto, Louis Waeckel, Stéphanie Longet, Sylvie Pillet, Elisabeth Botelho-Nevers, Manuel Rosa-Calatrava, Thomas Bourlet, Stéphane Paul

**Affiliations:** a Team GIMAP, Univ Lyon, Université Claude Bernard Lyon 1, Inserm, U1111, CNRS, UMR530, CIC 1408 Vaccinology, CIRI – Centre International de Recherche en Infectiologie, Saint-Etienne, France; b Team VirPath, Université de Lyon, INSERM U1111, Université Claude Bernard Lyon 1, CNRS, UMR5308, ENS de Lyon, CIRI - Centre International de Recherche en Infectiologie, Lyon, France; c VirNext, Faculté de Médecine RTH Laennec, Université Claude Bernard Lyon 1, Université de Lyon, Lyon, France; d Infectious Agents and Hygiene Department, University Hospital of Saint-Etienne, Saint-Etienne, France; e Immunology Department, University Hospital of Saint-Etienne, Saint-Etienne, France; f Infectious Diseases Department, University Hospital of Saint-Etienne, Saint-Etienne, France; g CIC 1408 Inserm Vaccinology, University Hospital of Saint-Etienne, Saint-Etienne, France; h Lead contact

**Keywords:** IgA, COVID-19, severity, lung, polyfunctionality

## Abstract

Several studies have investigated the antibody response to SARS-CoV-2, focusing particularly on the systemic humoral immune response and the production of immunoglobulin G (IgG) antibodies. IgA antibodies play a crucial role in protecting against respiratory viral infections but have also been associated with the pathophysiology of COVID-19. We performed a prospective study of 169 COVID-19 patients – 50 with critical/severe (ICU), 47 with moderate (Non-ICU), and 72 with asymptomatic COVID-19 – to explore the humoral immune response to SARS-CoV-2 infection. We found that the early systemic IgA response strongly induced in patients with severe disease did not block IgG neutralization functions and activated FcRs more effectively than IgG. However, even if SIgA levels were high, mucosal IgA antibodies could not control the infection effectively in patients with severe disease. Our findings highlight the complexity of the immune response to SARS-CoV-2 exhibiting high systemic levels of IgA with strong neutralizing capacity in severe cases, together with higher levels of IgA-FcR activation than in asymptomatic patients. They also suggest the need for further research to fully understand the role of IgA and its structural alterations in mucosal tissues in cases of severe disease and the impact of these antibodies on disease progression.

## Introduction

The COVID-19 pandemic due to severe acute respiratory syndrome coronavirus 2 (SARS-CoV-2) continues to pose significant global health challenges [1]. Several studies have investigated the antibody response to SARS-CoV-2, focusing particularly on the systemic humoral immune response and the production of immunoglobulin G (IgG) antibodies [2–4]. The licensed vaccines are administered intramuscularly, generating a systemic immune response but very weak mucosal immunity, whereas a mucosal immune response would be more appropriate for a respiratory virus such as SARS-CoV-2 [5,6]. IgA plays a crucial role in protecting against respiratory virus infections. For instance, the IgA antibodies present in nasal secretions have been found to neutralize respiratory viruses efficiently, such as influenza and respiratory syncytial virus (RSV) [7,8]. Recent studies have shown that IgA antibodies against SARS-CoV-2 can be detected in the saliva, tears, and nasal secretions of infected individuals, suggesting that they may play a role in preventing the transmission of the virus via these routes; however, these antibodies wane after nine months of hospitalization and they are not induced by subsequent vaccination [6,9–12]. Moreover, patients with selective IgA deficiency (sIgAD) have been shown to have higher rates of infection with SARS-COV-2 than patients without this deficiency, and higher rates of severe COVID-19 development [13,14]. Conversely, IgA has also been associated with the pathophysiology of COVID-19. Persistent spike-specific IgA responses in BAL samples collected from patients in the later stages of the infection have been shown to be associated with mortality [15]. Furthermore, IgA–virus immunocomplexes (ICs) potentiate the programmed cell-death pathway through which neutrophils release neutrophil extracellular traps (NETosis), via Fc-αRI engagement [16]. Aberrant patterns of IgG glycosylation are associated with a lack of FcR-dependent functions in patients with severe COVID-19, and thus with a more inflammatory immune response [17–19], but little is known about the FcR-dependent functions of IgA and their contribution to pathological effects in COVID-19.

In this study, we investigated mucosal and systemic humoral immunity in a cohort of COVID-19 patients, focusing on the specific contributions of IgA and IgG antibodies to disease severity. We therefore explored the dynamics and functionality of IgA and IgG antibodies in patients with severe COVID-19 patients, comparing these patients with those displaying milder symptoms or remaining asymptomatic.

## Results

### Early high levels of systemic IgA are associated with severe COVID-19

We performed a prospective study to investigate the humoral immune response to SARS-CoV-2 infection in 169 COVID-19 patients who were investigated during the first wave of the pandemic (first semester of 2020) and gave informed consent: 50 with critical/severe [hospitalized in an intensive care unit (ICU)], 47 with mild/moderate (hospitalized but not in the ICU), and 72 with asymptomatic disease (see the cohort description in the Materials and Methods section). We first determined total IgG1, IgG2, IgG3, IgG4, IgA, and IgM antibody levels in serum samples with the Bio-Plex immunoassay (BioRad Inc.) (Supp. Figure 1). During the first 10 days after the onset of symptoms, antibody levels did not differ between patients hospitalized in the ICU (ICU patients) and those hospitalized in departments other than the ICU (Non-ICU patients). Total antibody levels cannot distinguish early-stage severe disease patients. However, from 10 days after symptom onset, ICU patients had higher serum levels of IgA than Non-ICU and asymptomatic patients [ICU: 2.0330 g/L *vs.* Non-ICU: 0.8357 g/L (*p *< 0.0001); *vs* asymptomatic: 0.5520 g/L (*p *< 0.0001)]. These differences were maintained 40 days after the onset of symptoms, when IgA levels had declined but remained significantly higher in the ICU patients [ICU: 1.6250 g/L *vs.* Non-ICU: 0.3000 g/L (*p *= 0.0420); *vs.* asymptomatic: 0.5222 g/L (*p *= 0.0036)]. These results suggest that IgA is induced earlier than other antibody isotypes in patients with the most severe disease and that IgA dominates the specific humoral response to SARS-CoV-2 in these patients.

**Figure 1.**
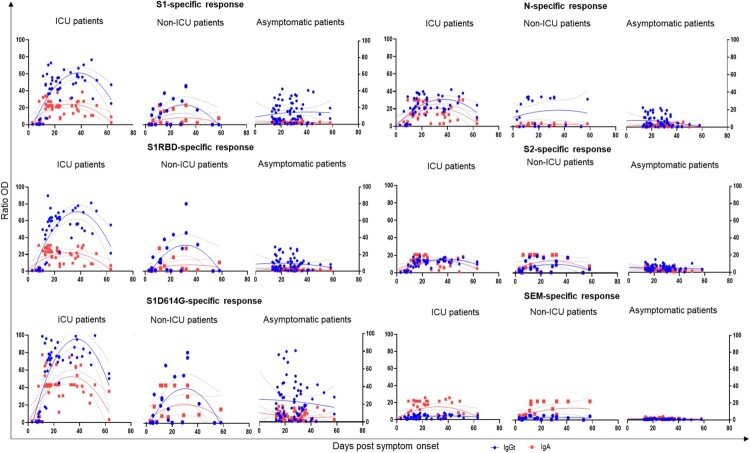
Kinetics of the antibody response in SARS-COV-2 infection. Specific anti-SARS-CoV-2 IgA and IgG antibody responses in serum directed against spike-1 protein (S1), spike-1 receptor-binding domain (RBD), D614G mutated S1 protein (S1D614G), nucleocapsid protein (N), spike-2 protein (S2), and polyprotein spike-envelope-membrane protein (SEM), at different times post-symptom onset. Each dot corresponds to one sample. Red squares correspond to IgA and blue circles to IgG. The dotted lines represent the nonlinear fit obtained by least squares regression with a 95% confidence interval.

We then investigated the kinetics of the SARS-CoV-2 specific antibody responses recognizing the spike 1 subunit (S1), the receptor-binding domain (RBD), the S1 mutated D614G, (S1-D614G), the nucleocapsid (N), the spike 2 subunit (S2), and the polyprotein before cleavage (SEM), in serum samples from the same cohort of patients (Figure 1). Stronger IgA and IgG responses were systematically observed in patients with severe disease, for all the targeted antigens. This pattern is clearer for all the S1 antigens (S1, RBD, and S1D614G), confirming that the spike protein is the most immunogenic of the viral proteins. Both IgA and IgG responses continued to increase until 40 days after the onset of symptoms in ICU patients, declining thereafter. By contrast, for Non-ICU patients, these responses peaked at about 30 days after symptom onset. These findings indicate that the specific antibody response to SARS-CoV-2 in the patients with the most severe disease is stronger and persists for a longer period of time than that in patients with less severe disease.

### The early systemic IgA response strongly induced in ICU patients does not block IgG functions

**Figure 2.**
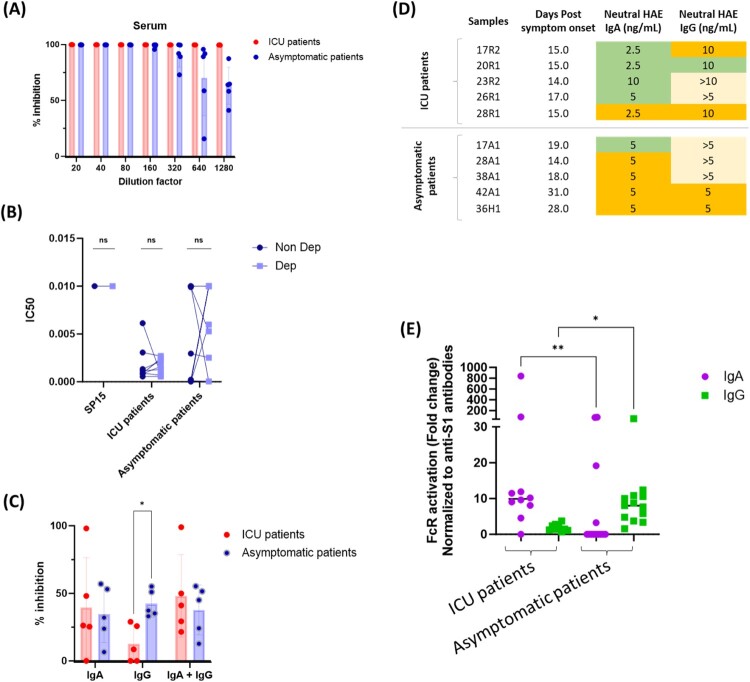
Effector functions of systemic anti-SARS-CoV-2 IgA and IgG antibodies. (A) Neutralizing activity of 10 sera tested at different dilutions in a live virus neutralization test. Each dot corresponds to a sample. (B) Neutralizing activity of IgA-depleted and non-depleted sera from 9 ICU patients and 11 asymptomatic patients, tested at equivalent IgG concentrations. SP15 corresponds to a pool of serum samples collected before the pandemic. *P* values were calculated with Sidak correction for multiple comparisons, with *α* = 0.05. (C) Neutralizing activity of purified IgA and IgG antibodies at a concentration of 10 μg/mL from 10 serum samples (5 ICU patients and 5 asymptomatic patients) collected between 15 and 30 days post-symptom onset. *P* values were calculated with Sidak correction for multiple comparisons, with *α* = 0.05 (*P < 0.05). (D) The neutralizing activity of purified IgA and IgG antibodies from 10 patients was measured in reconstituted human airway epithelia (HAE). Green shading indicates complete neutralization, orange shading indicates partial neutralization and yellow shading indicates an absence of neutralization. (E) Fold-change of FcR activation capacity dependent on CD89 (IgA) or CD16 (IgG) for serum samples from 23 patients tested at a 1:100 dilution in vitro. Each dot corresponds to one sample. *P* values were calculated with Dunn’s correction for multiple comparisons, with *α* = 0.05 (*P < 0.05, *P < 0.01).

We investigated the functionality of the humoral immune response elicited during infection by assessing the neutralizing activity of the antibodies against the Wuhan strain in a live virus neutralization test *in vitro*. Serum samples from the ICU patients neutralized the virus even at the last dilution tested (1:1280), whereas neutralizing activity began to decline after the 1:320 dilutions for serum samples from asymptomatic patients (Figure 2A).

As IgA and IgG levels were elevated at the beginning of the infection, especially in the most severe patients, we then explored whether these antibodies contributed equally to the serum neutralizing activity across different patient groups and whether high systemic IgA levels could competitively inhibit IgG functions. To test this, we depleted total IgA antibodies from the serum samples of nine ICU patients and eleven asymptomatic patients using agarose beads coupled to Peptide M. This depletion was more than 90% effective for all the samples (Supp. Figure 2). We then evaluated the neutralizing activity of samples with and without IgA depletion in the same live virus neutralization assay. Our results showed no significant differences between ICU and asymptomatic patients before and after IgA depletion, suggesting that IgA did not inhibit IgG's potential neutralizing effects (Figure 2B).

We further investigated the individual contributions of IgA and IgG to SARS-CoV-2 neutralization, by purifying IgA and IgG from the serum samples of 10 patients (5 ICU and 5 asymptomatic patients) by affinity chromatography with Peptide M-coupled beads and Protein G-coupled beads, respectively. The antibody preparations obtained had a purity of more than 98% (Supp. Figure 3), and we systematically checked for the presence of residual IgG or IgA (data not shown). We assessed the neutralization potential of the purified antibodies at a concentration of 10 µg/mL in the same *in vitro* neutralization test. There was no significant difference in IgA neutralization activity between ICU and asymptomatic patients (Figure 2C). By contrast, purified IgG from ICU patients had a lower neutralizing capacity (13% inhibition) than IgG from asymptomatic patients (42% inhibition). This difference was statistically significant and suggests that the neutralization capacity of IgG antibodies is impaired in patients with the most severe disease. When purified IgA and IgG were mixed, neutralization did not differ between ICU and asymptomatic patients. The loss of IgG neutralization function does not, therefore, affect overall neutralization rates when IgA is also present, corroborating that there is no competition or blocking effect between IgA and IgG. We wanted to evaluate the neutralizing activity of purified IgA and IgG in the highly predictive and physiologically relevant MucilAir^TM^ reconstituted human airway epithelium (HAE) model (Figure 2D). The incubation of SARS-CoV-2 with 2.5-10 ng/mL purified IgA from patients with severe disease resulted in complete viral neutralization in four of the five samples tested, and partial neutralization (>2 log_10_ decrease in viral titer in the first 48 h but with viral replication still measurable) in the remaining sample. Higher levels of purified IgG from the same patients were required to achieve complete neutralization for one of the five tested samples and partial neutralization for another two. For asymptomatic patients, complete and partial neutralization was achieved for one in five and four in five of the samples tested for purified IgA, whereas partial neutralization was achieved for only two of the five samples tested with purified IgG. Overall, these results corroborate the *in vitro* neutralization results that IgA from severe disease patients have higher neutralizing potential than those from less severe patients, and that there is a loss of neutralizing function for the IgG of these patients.

As neutralization is not the only effector function antibodies can do to fight against viruses, we further assessed the Fc receptor potential and ADCC-like functions of systemic IgA and IgG. For this, we employed a previously described *in vitro* test based on the use of HEK cells expressing either CD16 or CD89 as effector cells [20]. IgA from ICU patients generated an FcR-dependent signal more efficiently than IgA from asymptomatic patients (mean relative FcR activation fold-change: 33 *vs.* 15; *P *< 0.01). The opposite pattern was observed for IgG (mean relative FcR activation fold-change: 16 *vs.* 30; *P *< 0.05), again suggesting a loss of IgG function in the serum of patients with severe disease and a gain of IgA function in the serum of patients with severe disease relative to asymptomatic patients (Figure 2E).

### Mucosal IgA antibodies do not control infection effectively in severe patients

**Figure 3.**
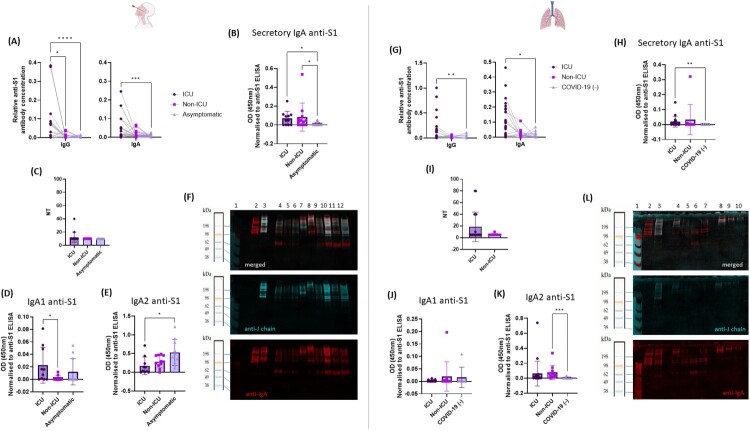
Mucosal immune response to SARS-CoV-2 in nasal swabs (A-F) and BAL (G-L). (A and G) Relative IgG and IgA anti-S1 concentrations, normalized to total IgG or IgA levels in 78 patients (A, nasal swabs) and 57 patients (G, BAL). Each dot corresponds to a separate sample. *P* values were calculated in Kruskal-Wallis tests with Dunn’s correction for multiple comparisons, with *α* = 0.05 (*P < 0.05, **P < 0.01, ***P < 0.001, and ****P < 0.0001). (B and H) Secretory anti-S1 IgA levels, normalized relative to anti-S1 IgA concentrations, in 43 patients (B, nasal swabs) and 34 patients (H, BAL). Each dot corresponds to a separate sample. *P* values were calculated in Kruskal-Wallis tests with Dunn’s correction for multiple comparisons, with *α* = 0.05 (*P < 0.05, **P < 0.01). (C and I) Neutralizing activity of 55 nasal swabs and 29 BAL tested at 1:10 and 1:5 dilutions, respectively, in a live virus neutralization test *in vitro*. (D and J) Anti-S1 IgA1 levels normalized relative to anti-S1 IgA concentrations; in 34 patients (D, nasal swabs) and 40 patients (J, BAL). Each dot corresponds to a separate sample. *P* values were calculated in Kruskal-Wallis tests with Dunn’s correction for multiple comparisons, with *α* = 0.05 (*P < 0.05). (E and K) Anti-S1 IgA2 levels, normalized relative to anti-S1 IgA concentrations, in 34 patients (E, nasal swabs) and 40 patients (K, BAL). Each dot corresponds to a separate sample. *P* values were calculated in Kruskal-Wallis tests with Dunn’s correction for multiple comparisons, with *α* = 0.05 (*P < 0.05, **P < 0.01, ***P < 0.001). (F and L) Western blot with immunofluorescence detection for an SDS-PAGE gel performed in non-reducing conditions on crude nasal swabs (F) (lane 1: molecular weight marker; lane 2: IgA control without chain J; lane 3: IgA control with chain J; lane 4-6: nasal swabs from 3 asymptomatic patients; lane 7–9: nasal swabs from 3 Non-ICU patients; lane 10-12: nasal swabs from 3 ICU patients); and BAL (L) (lane 1: Molecular weight marker; lane 2: IgA control without chain J; lane 3: IgA control with chain J; lane 4-7: BAL from 4 ICU patients; lane 8-10: BAL from 3 Non-ICU patients) with the detection of IgA monomers and dimers. For all the samples, a total amount of 20 μg of protein was loaded.

As SARS-CoV-2 is a respiratory virus, we investigated the humoral immune response in the mucosa. We compared anti-SARS-CoV-2 IgA and IgG responses in nasal swabs and bronchoalveolar lavages (BAL) from the same patients, with normalization against the total of IgA or IgG antibodies concentration in the sample. The anti-SARS-CoV-2 S1 IgG response in nasal swabs was stronger in ICU patients than in Non-ICU (relative antibody concentration mean ranks: 58.34 *vs.* 40.84; *p *< 0.05) or asymptomatic patients (relative antibody concentration mean ranks: 58.34 *vs.* 27.99; *p *< 0.0001) (Figure 3A). ICU patients also had a stronger nasal S1 IgA response than asymptomatic patients (relative antibody concentration mean ranks: 55.83 *vs.* 30.25; *p *< 0.001) (Figure 3A). In BAL samples significant differences were found only between ICU and SARS-CoV-2-negative patients (replacing the asymptomatic patients, who did not undergo BAL, for this analysis) for IgA (relative antibody concentration mean ranks: 35.30 *vs.* 18.45; *p *< 0.05) and IgG (relative antibody concentration mean ranks: 34.14 *vs.* 17.33; *p* < 0.01) (Figure 3G).

We then compared levels of anti-spike specific secretory IgA (SIgA) between the three groups of patients with a newly developed assay. In both mucosal compartments (nasal swabs and BAL), SIgA levels were higher in ICU and in Non-ICU patients compared to asymptomatic patients ([nasal swabs, mean ranks: ICU: 27.36; Non-ICU: 26.92; Asymptomatic: 16.10] and [BAL, mean ranks: ICU: 22.16; Non-ICU: 11.11; SARS-CoV-2-negative: 8.000]), and the differences were statistically significant (Figure 3B,H). Surprisingly, we found no neutralizing activity in the nasal swabs or BAL samples for any of the groups (Figure 3C,I). As IgA2 is the most predominant IgA isotype in mucosa, we investigated the levels of IgA1 and IgA2 specific to SARS-CoV-2 between groups in nasal swabs (Figure 3D,E) and in BAL (Figure 3J,K), given it has been previously described that IgA isotypes could have differential roles in inflammation and activation of immune cells [21–23]. Nasal swabs from ICU patients contained significantly higher levels of anti-S1 IgA1 than nasal swabs from Non-ICU patients (nasal swabs, mean ranks: ICU: 21.60; Non-ICU: 12.36; *P *< 0.0339) (Figure 3D). For BAL samples, we found no significant difference between the groups (Figure 3J). However, we did find significant differences in anti-S1 IgA2 in nasal swabs between ICU patients and asymptomatic patients but not between ICU and Non-ICU patients (nasal swabs, mean ranks: ICU: 10.10; Non-ICU: 16.09; Asymptomatic: 21.80; ICU vs. Asymptomatic: *P *< 0.0339) (Figure 3E). For BAL samples, significant differences were found between Non-ICU patients and SARS-CoV-2-negative patients (BAL mean ranks: Non-ICU: 28.55; Asymptomatic: 8.714; *P *< 0.0009) (Figure 3K). Despite the lack of a significant difference between ICU and Non-ICU patients, there was a trend towards lower anti-S1 IgA2 levels in the patients with the most severe disease, suggesting an impairment of the local immune response in ICU patients. The IgA in mucosal tissues is usually dimeric, and monomeric IgA might not provide adequate protection against viral infections in these tissues [24]. We therefore checked for any changes to the structure of the IgA present in the mucosal samples studied. Both monomers and dimers were detected in nasal swabs from all three groups (Figure 3F). However, monomer concentrations were significantly higher in the BAL from ICU patients, but not in those from Non-ICU patients (Figure 3L).

## Discussion

We studied the humoral immune response to SARS-CoV-2 in a cohort of patients from the first wave of the COVID-19 pandemic in France in 2020. We found that systemic IgA levels were significantly higher in ICU patients than in Non-ICU or asymptomatic patients. This response was more robust and persistent in severe COVID-19 cases, and was polyreactive as well as specific to the S1, RBD, S2, and N proteins of SARS-CoV-2, as previously described [4,15]. These findings support previous studies showing that patients with severe COVID-19 have a more pronounced and persistent antibody response [9,17,25–31], partly due to the persistence of SARS-CoV-2 proteins and mRNA in the small intestine epithelium even months after infection [32–34]. In addition, high levels of IgA early in the disease (10-15 days after the onset of symptoms) were reported in a previous study, which found that the CCR10^+^ plasmablasts circulating at this stage of the disease secreted predominantly IgA [9]. Such observations suggest that the early presence of anti-spike IgA in the bloodstream could potentially serve as an early indicator of disease severity and that these antibodies play a pivotal role in the immune response to SARS-CoV-2.

The high levels of serum IgA as early as one week after symptom-onset in the more critical patients led us to hypothesize that IgA might block IgG functions, as it has been described for other viral infections like HIV [35]. However, in our *in vitro* models with VeroE6 cells and with the lung epithelium model, we found that systemic IgA from severe patients neutralized the SARS-CoV-2 virus more effectively than IgG, and also more effectively than IgA and IgG from asymptomatic patients. This suggests that there is no competition between IgA and IgG for neutralization and that IgA is a more potent SARS-CoV-2 neutralizer than IgG. These findings align with previous studies [36,37] which reported that serum samples from patients with severe disease neutralize the infection more effectively *in vitro* than serum samples from asymptomatic patients. However, contrary to a previous study [38], we found no difference in neutralization levels before and after IgA depletion. This discrepancy may stem from differences in the methods used to assess viral neutralization. While Davis *et al*. used an RBD-ACE2 binding inhibition multiplex bead-based assay, we employed an *in vitro* neutralization test that more closely mimics real-life conditions and is a more complex system than one based solely on RBD-ACE2 binding inhibition. Furthermore, consistent with prior research [9], we observed that IgA purified from the serum of patients with severe disease had a higher neutralization potential than IgA from asymptomatic patients, particularly in a more physiological lung epithelium model . Conversely, IgG purified from patients with severe disease had weaker neutralizing activity than IgG from asymptomatic patients. This result is consistent with other reports of a loss of IgG function associated with impaired neutralization activity in patients with severe COVID-19 [9,17]. Importantly, there was no evidence to suggest that IgA blocked the neutralizing effect of IgG as observed in other infections, such as HIV [35]. As proposed in other studies, these results suggest instead that neutralizing IgA activity may play a crucial role in the acute phase of the infection [39].

However, neutralizing activity is not the only functional property of antibodies during a viral infection. FcR-dependent functions can also contribute to disease control and promote a robust immune response. In our study, we observed that the CD16-dependent function of IgG was weaker in patients with severe disease than in asymptomatic patients. These findings contrast with other studies that report higher levels of FcR activation for IgG from patients with severe disease. These studies also found that IgG antibodies produced in severe patients are structurally different compared to those in asymptomatic patients [17–19,40,41]. These antibodies are less fucosylated and sialylated, making them more active in some FcR-dependent functions like ADCC and ADCD, leading to increased immune system activation and inflammation [17–19,40,41]. This phenomenon has also been described for other pathologies like MS (multiple sclerosis) and RA (rheumatoid arthritis) [21]. Contrary to these studies, our findings show a loss of CD16-dependent function in the serum from severe patients compared to asymptomatic patients. These findings contradict those studies which have reported an increased FcR activation in sera from severe patients, associated with afucosylated IgG which has an enhanced FcR affinity [17,42]. Further investigation is needed to determine whether the observed loss of function in our study is due to receptor affinity, or structural differences between IgG from severe and asymptomatic patients. Recently a study showed that serum IgM antibodies in severe COVID-19 patients have also a distinct glycosylation profile compared to moderate disease patients [43]. This study found increased di- and tri-sialylated glycans and altered mannose glycans in total serum IgM in severe patients, as well as elevated levels of antigen-specific IgM ADCD [43]. While the FcR-dependent functions IgG and IgM in COVID-19 have been studied, less is known about the IgA-specific FcR-dependent functions. Here we demonstrated that serum IgA antibodies from severe patients have higher CD89 activation compared to asymptomatic patients. [17,42]. However, another study reported no effect of IgA depletion on antibody-dependent phagocytosis (ADP), suggesting that IgA contributes to neutralization but does not efficiently activate certain FcR-dependent functions [38]. By contrast, our findings are consistent with previous reports of higher levels of NETosis triggered by IgA in patients with severe disease [44,45]. Moreover, IgA may also have undergone structural modifications in patients with severe disease, enhancing its FcR-dependent activities. To date, no studies have explored the glycosylation profile or post-translation modifications in the IgA antibodies of COVID-19 patients, which could provide insights into the pathological role of this antibody in SARS-CoV-2 infection. It would be valuable to conduct this study in the near future, as it has been shown, for example, in RA that modifications in the glycosylation profile of IgA in serum can modify effector functions, potentiating NETosis and thereby increasing inflammation [21]. In COVID-19, it is known that higher levels of neutrophils and NETosis induction occur, as well as longer persistence of NETs in tissues and bloodstream, with IgA possibly contributing to this hyperactivation of neutrophils [44–46]. In addition, IgA glycosylation modifications can increase complement deposition and the formation of inclusion bodies, as is the case in the IgA nephropathy [47]. Small-vessel vasculitis driven by deposits of IgA-C3 ICs [48], or IgA vasculitis, formerly known as Henoch–Schönlein purpura (HSP), has also been reported in children with COVID-19, suggesting that SARS-CoV-2 infection may be a trigger [49]. Taken together, our results show that neutralization rates are higher for systemic IgA than for IgG, particularly in patients with severe disease, and that IgA-FcR activation is stronger in patients with severe disease than in asymptomatic patients. However, this uncontrolled IgA activation and the compromised functionality of IgG antibodies in the early phase of the disease may exacerbate the inflammatory response, contributing to the severity of the disease, as widely described elsewhere [17,50].

We also investigated mucosal immune responses, specifically in nasal swabs and BAL samples. As reported in previous studies [29,51,52], we observed higher levels of anti-SARS-CoV-2 IgA and IgG in nasal swabs and BAL from ICU patients compared to those from asymptomatic or SARS-CoV-2-negative patients, indicating a more robust immune response in severe cases. However, the lack of neutralizing activity in both nasal swabs and BAL samples across all patient groups contradicts the findings of other studies reporting neutralizing activity in mucosal samples [9,53,54]. These other studies assessed SARS-CoV-2 neutralization using a pseudovirus *in vitro*. However, despite the advantages of tests of this type [55], the test used in these previous studies focused exclusively on the spike protein of SARS-CoV-2 and therefore provided a less complete picture than the live virus neutralization test used here. Ruiz, *et al*. reported a loss of neutralizing activity in BAL collected during the later stages of COVID-19 from patients who did not survive, despite the persistence of S1-, RBD-, S2-, and NP-specific IgG and S1-specific IgA after SARS-CoV-2 had been cleared from the lungs [15]. These findings suggest that the high levels of IgA found here, even if not-neutralizing, could activate FcR-dependent functions, as suggested by Ruiz, *et al*., via the formation of IgA immunocomplexes (ICs) [15]. We also observed higher levels of sIgA in patients with severe disease compared to individuals testing negative for SARS-CoV-2; sIgA may promote lung inflammation and fibrosis in humans by inducing fibroblast activation and increasing the production of inflammatory cytokines [56]. The IgA present in mucosal tissues is mostly dimeric and locally produced, but plasma monomeric IgA can reach the airways through a receptor-independent process called transudation [57,58]. This process is more likely to occur in damaged lung tissue, as seen in patients with severe COVID-19. We detected both monomers and dimers of IgA in mucosal secretions, but with the balance in favour of monomers in BAL from patients with severe disease, as reported by Sterlin *et. al* [9]. IgA-dependent FcR activation was stronger in the serum of patients with severe disease. If the IgA monomers found in mucosal tissues arrived through transudation from the serum, it would be expected that FcR function, and consequently inflammation, would be greater in mucosal tissues. Moreover, mIgA1 has been reported to activate cytotoxic CD8^+^ T cells [23], and an aberrant glycosylation profile has been reported to increase the pro-inflammatory effect of these molecules, leading to greater disease severity in autoimmunity [21,59]. Although the glycosylation pattern of IgA antibodies in COVID-19 has yet to be studied in detail, aberrant glycosylation has been reported for IgG in this context.

Our results suggest that the mucosal antibody response may be less effective at controlling the infection than previously thought, at least in terms of neutralization, and that FcR-dependent function may contribute to the pathophysiology of the disease in the upper and lower respiratory tract. The contrasting findings in our study and the discrepancies with other studies highlight the complexity of the mucosal immune response to SARS-CoV-2, suggesting variability in the immune profiles across different patient populations.

Our study is subject to several limitations as it was a prospective study performed on samples collected during the early wave of the pandemic where the Wuhan SARS-CoV-2 strain was the only one circulating. This may reduce the relevance of the findings to current and future variants, however, that first wave of infections is very interesting as all the infected patients were naïve and we saw in the clinic that with the new variants there were less severe cases of COVID-19, because of the previous immunity to SARS-CoV-2. Furthermore, a Wuhan patient cohort is very useful to decipher the pathological role of antibodies in the pathophysiology of the COVID-19 disease. Additionally, the limited number of samples, especially for the IgA and IgG neutralization assays, constrains the power of the study, limiting the generalizability of our findings. Unfortunately, in this study we do not have a long-term follow-up data, which could help to understand the persistence and long-term effects of the IgA response systemically and in mucosal tissues. Furthermore, the low levels of IgA and IgG antibodies found in the serum of asymptomatic patients precluded a comprehensive quantitative exploration of all the functional and structural properties of SARS-CoV-2-specific antibodies in our patient cohort. Moreover, it remains to be determined whether the antibodies present in mucosal secretions contribute to the pathology of COVID-19 through FcR-functions.

In conclusion, this study highlights the complex and varied immune response to SARS-CoV-2, with high systemic levels of IgA with strong neutralizing capacity in severe cases, and higher levels of IgA-FcR activation observed for IgA from patients with severe disease than for IgA from asymptomatic patients. However, the contradictory findings regarding neutralization and FcR-dependent functions in mucosal tissues underscore the need for further studies to fully understand the role of IgA antibodies, their structural alterations in severe cases, and their impact on disease progression.

## MATERIALS and methods

### Study design

We included 169 PCR-positive SARS-CoV-2-infected patients in a prospective cohort study conducted at CHU Nord de Saint-Etienne (Saint-Etienne, France) during the first wave of SARS-CoV-2 infections in France (from February 2020 to May 2020). COVID-19 patients were split into three groups according to the WHO classification of disease severity [60]: 72 patients with mild or asymptomatic disease, 47 patients who were hospitalized but did not require admission to the ICU, and 50 patients with severe disease requiring admission to the ICU (Table 1). Serum samples were collected between 1 and 67 days post symptom onset. Nasal swabs were collected at the time of the first PCR for the confirmation of SARS-CoV-2 infection. Bronchoalveolar lavage was collected whenever possible for hospitalized patients (ICU and Non-ICU) and from 16 patients testing negative by PCR for SARS-CoV-2 sampled before the pandemic. Written informed consent for participation was obtained from all subjects, and ethics approval was obtained from CPP Ile de France V (NCT04648709).

**Table 1.** Patients’ characteristics per clinical group.

Characteristics	Patient's group		
	ICU patients (*n* = 50)	Non-ICU patients (*n* = 47)	Asymptomatic patients (*n* = 72)
Sex			
Female	10	18	53
Male	40	29	19
Age (years), median (IQR)	70	76	33.5
Comorbidities (%)			
Age >70	54	34	–
Obesity	16	10.6	–
Hypertension	48	31.2	–
Diabetes	26	10.6	–
Cardiovascular disease	8	6.4	–
Kidney failure	6	2.1	–
Chronic respiratory disease	16	2.1	–
Malignancy	4	8.5	–

IQR: Interquartile range.

### Cells

VeroE6 cell lines were obtained from American Type Culture Collection (ATCC), CRL-1586, (not authenticated but regularly tested for mycoplasma contamination). HEK CD89^+^ and HEK CD16^+^ cell lines were obtained from InvivoGen and used as effector cell lines for the FcR activation assay. All cells were maintained in Dulbecco′s modified Eagle medium (DMEM) supplemented with antibiotic-antimycotic (AAT) and 2% SVF (for VeroE6 cells) or 10% SVF (for HEK CD89^+^ and HEK CD16^+^ cells). Cells were harvested with trypsin/EDTA solution. All cell lines were maintained at 37°C under a humidified atmosphere containing 5% CO_2_.

HEK-CD89^+^ (FcαRI) and HEK-CD16^+^ (FcγRIIIa) cell lines were obtained from InvivoGen and have been described elsewhere [20]. All cells were maintained in DMEM supplemented with 10% FCS and 1% antibiotic-antimycotic (AAT). Cells were harvested with trypsin/EDTA solution and the stable expression of FcR was checked by flow cytometry before each experiment. All cell lines were maintained at 37°C, in a humidified atmosphere containing 5% CO_2_.

### Quantification of total immunoglobulins

Serum samples were tested with the Bio-Plex Pro Human Isotyping Panel assay (Bio-Rad Laboratories, Hercules, CA) according to the manufacturer’s instructions. Briefly, 50 μL diluted beads were added to each well, and plates were washed with Bio-Plex wash buffer. Standards, blanks, controls, and samples were added at the indicated dilutions and the plates were incubated at 25°C for 1 h. Another washing step was performed, and the detection antibodies were added. The plates were then incubated for 30 min at 25°C in the dark. Plates were washed and streptavidin-PE diluted 1X was added and incubated with the plates for 10 min at 25°C in the dark. A final washing step was performed, and the beads were resuspended in 125 μL assay buffer. Plates were read in a Bio-Plex Luminex 200 system. The results are expressed in g/L.

### Measurement of spike-specific IgA

IgA binding to the SARS-CoV-2 spike protein was evaluated by ELISA. In brief, a spike protein solution (1 μg/mL; #40591-V08H Spike S1-RBD Sino Biologicals) was incubated with high-binding 96-half-well plates (Thermo Fisher Scientific) overnight at 4°C. The plates were washed and blocked, and serum samples were added and incubated for 1 h at 37°C. The plates were washed and then incubated with an anti-human IgA (A0295; Sigma-Aldrich) or IgG (A6029; Sigma-Aldrich) secondary Ab conjugated to horseradish peroxidase (HRP). The HRP substrate (3,3′,5,5″-tetramethylbenzidine (TMB; 34021; Thermo Fisher Scientific)) was added, followed by a stop solution (1 M HCl), and optical density was then measured at 450 nm in a microplate reader (TECAN). For serum samples, a ratio of OD values between the sample and negative control (pool of pre-pandemic serum samples) added in duplicate to each run was calculated. For nasal swabs and BAL samples, equivalent IgA and IgG content was calculated from a calibration curve with a recombinant anti-RBD IgA (B Cell Design #IB3C4 PV) or anti-RBD IgG (B Cell Design # X30F12-PU), with a limit of detection of 0.1 ng/mL equivalent.

### IgA depletion and immunoglobulin purification

IgA depletion was performed on 21 serum samples (from 9 ICU patients and 12 asymptomatic patients). We diluted 100 μL serum in 1× PBS and mixed the resulting solution with 100 μL peptide M/agarose column (InvivoGen). The mixture was loaded onto the column after resin equilibration. The columns were incubated for 1 h at 25°C and the IgA-depleted plasma fractions were collected by spinning the resin columns at 1000×*g* for 1 min. Purified IgA was eluted with 0.1 M glycine (pH 2 to 3; Sigma-Aldrich), and the pH was immediately adjusted to 7.5 with 1 M Tris.

We increased the amount of purified immunoglobulin (Ig) obtained by diluting 500 µL in 1× PBS and passing the resulting solution through a double chromatography system as follows: first, the diluted serum was loaded onto an equilibrated protein G/agarose column (InvivoGen). The flowthrough was collected and loaded onto a second column filled with peptide M/agarose (InvivoGen) to obtain purified IgA. IgA and IgG were eluted with 0.1 M glycine (pH 2 to 3; Sigma-Aldrich), and the pH was immediately adjusted to 7.5 with 1 M Tris. PBS buffer exchange was performed with Amicon Ultra centrifugal filters (Merck Millipore) with a 50 kDa membrane, in accordance with the manufacturer’s instructions. All chromatography steps were performed at a flow rate of 0.5 mL/min. The purity of the IgG and IgA fractions was evaluated by ELISA. The purified Ig preparations contained less than 1% undesirable isotypes.

### SARS-CoV-2 live virus neutralization assays in Vero E6 cells

For the detection of neutralizing antibodies and determination of their levels, we used a plaque reduction neutralization test (PRNT). Each serum was first diluted 10-fold in culture medium and heated for 30 minutes at 56°C to prevent complement-mediated decreases in viral activity. We then mixed two-fold serial dilutions of each serum sample in equal volumes with the live SARS-CoV-2 virus (strain: 19A (B.38)) and incubated the mixture for 1 h at 37°C. The mixture was transferred to 96-well microplates covered with Vero E6 cells to achieve a viral concentration of 100 TCID_50_/well and the plates were incubated at 37°C under an atmosphere containing 5% CO_2_. The microplates were examined under a microscope five days later and the number of wells displaying a cytopathic effect was determined. Viral quantification was performed by RT-PCR on the supernatant, as previously described [61] and the results obtained were used to calculate the % inhibition of infection. We used the same protocol for nasal swabs and BAL samples, except that the nasal swabs were first diluted 1:5, and BAL 1:10 in culture medium, and the neutralizing titer was determined as the last sample dilution resulting in the infection of 50% of the wells. For IgA depletion, the IC_50_ was calculated with GraphPad Prism 10.0.1 (GraphPad, San Diego, CA, USA). All experiments were performed in a biosafety level 3 laboratory.

### SARS-CoV-2 live virus neutralization assays in HAE

We evaluated the neutralization capacity of purified IgA and IgG from patients, by mixing 75 µL of each Ig sample (final concentrations of 2.5, 5, and 10 ng/mL) with 75 µL of a suspension of Wuhan-like 19A SARS-CoV-2 virus (GISAID accession number: EPI_ISL_411218; 19A (B)) at an MOI of 0.01. The mixture was incubated for 1 h at 37°C and was then used to inoculate the apical side of a MucilAir^TM^ nasal HAE (Epithelix SARL, ref: EP02, pool ref: MP0010) maintained at the air–liquid interphase. The apical poles of the HAE were washed gently twice with warm OptiMEM (1X) medium (GIBCO BRL, ref. 31985-047) before inoculation. The HAE was incubated for 1 h at 37°C under an atmosphere containing 5% CO_2_. Samples were tested in duplicate and recombinant anti-spike and non-SARS-CoV-2-specific IgA/G were used as positive and negative controls, respectively. One-hour post-inoculation (hpi), the Virus-Ig mixture was removed, and the HAE was returned to the incubator. The apical poles of the HAE were washed, at 24 hpi, with warm OptiMEM, which was then collected for the quantification of viral nsp14 gene copies (by RT-qPCR) as previously described [62]. Washing and quantification were repeated at 48 and 72 hpi.

### SARS-CoV-2 RT–PCR for viral amplification

The amount of SARS-CoV-2 in the culture supernatant was measured by RT–PCR without the need for nucleic acid extraction. The Luna Universal Probe One-Step RT-qPCR Kit from New England Biolabs (Ref. E3006L) [63] was used for this purpose. We diluted 5 μL supernatant 1/10 in DNase-free and RNase-free water. The resulting solution was mixed with the reaction solution to obtain a total volume of 14 μL. The reaction solution contained 5 μL Luna® Universal Probe One-Step Reaction Mix, 0.5 μL Luna® WarmStart® RT Enzyme Mix and 1.5 μL of a mixture of primers at 400 nM (E_Sarbeco_F: ACAGGTACGTTAATAGTTAATAGCGT and E_Sarbeco_R: ATATTGCAGCAGTACGCACACA) and the probe at a concentration of 200 nM (E_Sarbeco_P1: FAM-ACACTAGCCATCCTTACTGCGCTTCG-BBQ) [64]. RT–PCR was initiated with a reverse transcription step at 55°C for 10 min, followed by 40 cycles of denaturation at 95°C for 10s and annealing at 60°C for 60s. A viral standard curve was generated for each analysis.

### SARS-CoV2-specific FcR activation assay

SARS-CoV-2-specific FcR activation assays were performed as previously described [20] with the following modification: SARS-CoV-2-infected VeroE6 cells were used as target cells, which were incubated with serum samples from the patients diluted 1:100 in medium. Effector cells expressing the Fc receptor (HEK-CD16^+^ or HEK-CD89^+^) were cocultured with the target cells at a 1:1 effector/target ratio for 48 h at 37°C. Alkaline phosphatase activity in the culture supernatant was assessed with Quantiblue (InvivoGen) at a wavelength of 620 nm. FcR activation was calculated as follows: OD_620nm_ sample/ OD_620nm_ pooled prepandemic sera. It was then normalized according to the anti-S1 antibody concentration for each sample.

### Immunoblotting

Nasal swabs and BALs were centrifuged at 200 x *g* for 5 min, and total protein concentrations were determined in a Bradford assay. Samples were diluted in deionized water and then in NuPAGE LDS Sample buffer 4X (Ref: NP0008, Thermo Fisher Scientific) to achieve a final amount of 20 μg total proteins in each of the samples loaded onto the gel. The samples were heated at 70°C for 10 min. Proteins were separated by electrophoresis in 4 to 12% Bis-Tris polyacrylamide gels (Ref: NW04125BOX, Thermo Fisher Scientific) for 45 min at 200 V and the resulting bands were then transferred onto nitrocellulose membranes for western blot analysis. The membrane was blocked by incubation with 5% nonfat milk in 1× PBS for 16 h, and human IgA and chain J were then detected by incubation with an anti-IgA-PerCPVio700 antibody (Ref: 130-116-885 Miltenyi Biotec) used at a dilution of 1:2000 and an anti-chainJ-AF488 antibody (Ref: sc-133177 AF488, Santa Cruz Biotechnology, INC) used at a dilution of 1:2000 dilution, respectively, for 1 h at 25°C. Fluorescence was visualized with a camera system (iBright 1500, Thermo Fisher Scientific). All incubations were performed in 5% nonfat milk in 1× PBS and washes were performed in 0.1% Tween 20 in 1× PBS.

### Statistical analysis

Statistical difference was determined by two-way ANOVA with Tukey or Sidak correction for multiple comparisons, depending on the data set. When samples were not normally distributed, the nonparametric Kruskal–Wallis test was also used with Dunn’s correction for multiple comparisons. Significant *P* values are indicated as described: *P < 0.05, **P < 0.01, ***P < 0.001, and ****P < 0.0001. All statistical calculations were performed, and the graphs were generated with GraphPad Prism 10.0.1 (GraphPad, San Diego, CA, USA).

## Supplementary Material

Supplemental Material

Supplemental Material

Supplemental Material

## Data and materials availability

All data are available in the main text or the supplementary materials.
