# Supplementary figures and images for: Impaired mucosal IgA response in patients with severe COVID-19
Source: Emerg Microbes Infect. 2024 Oct 2;13(1):2401940. doi: 10.1080/22221751.2024.2401940 (PMC11451292; doi:10.1080/22221751.2024.2401940)

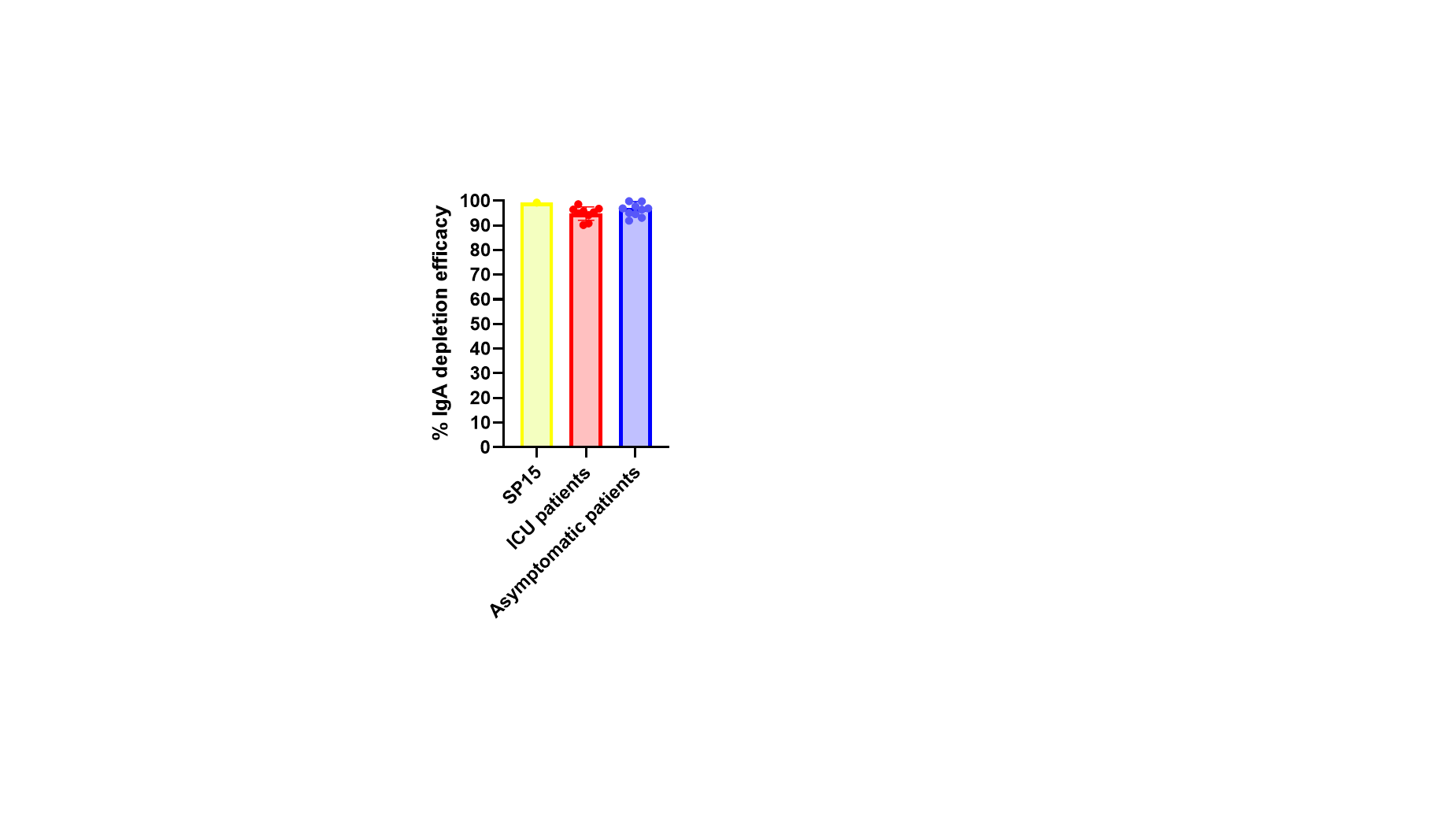

Supplement: Supplemental Material [file TEMI_A_2401940_SM3958.tiff]

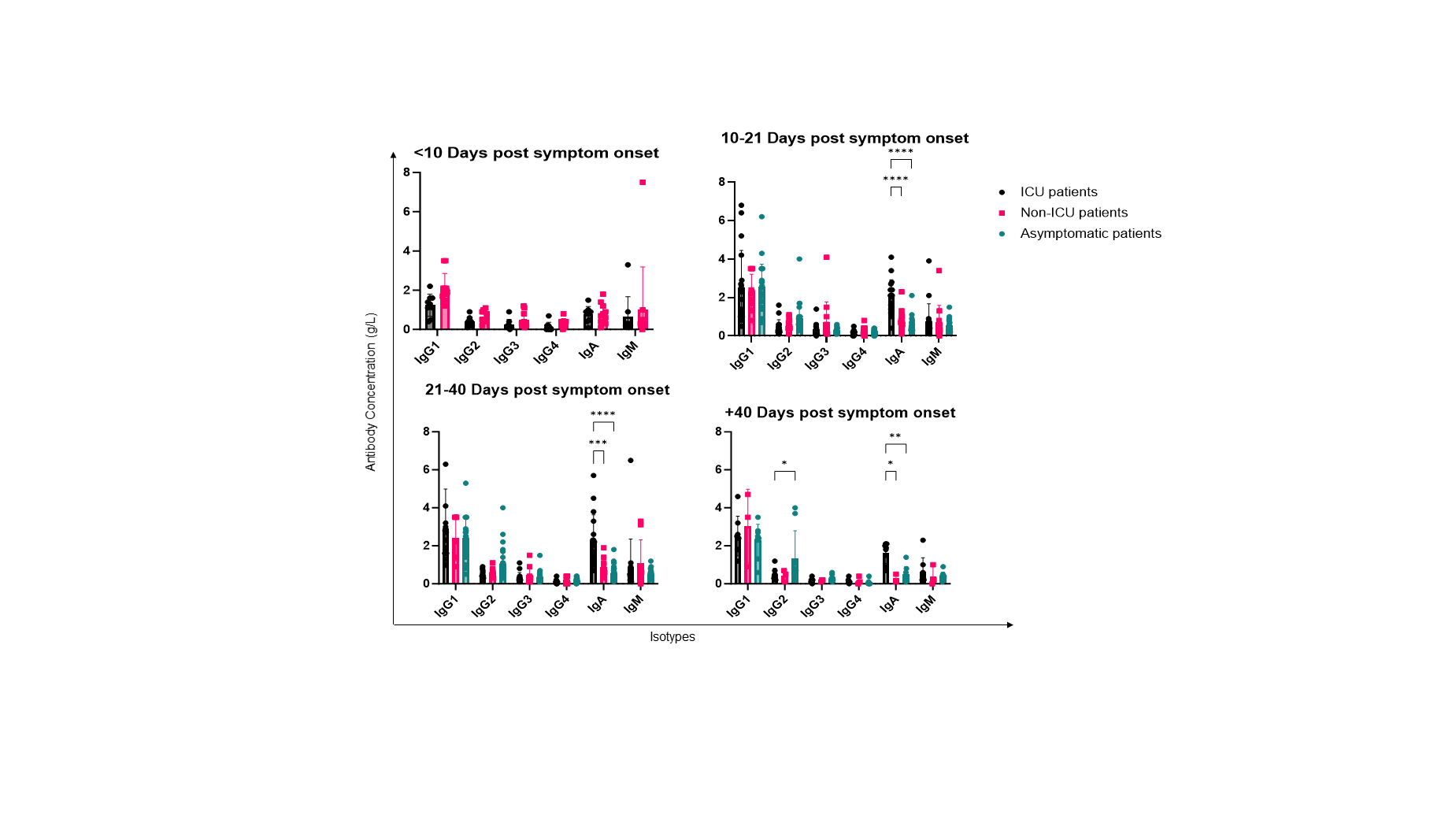

Supplement: Supplemental Material [file TEMI_A_2401940_SM3957.tiff]

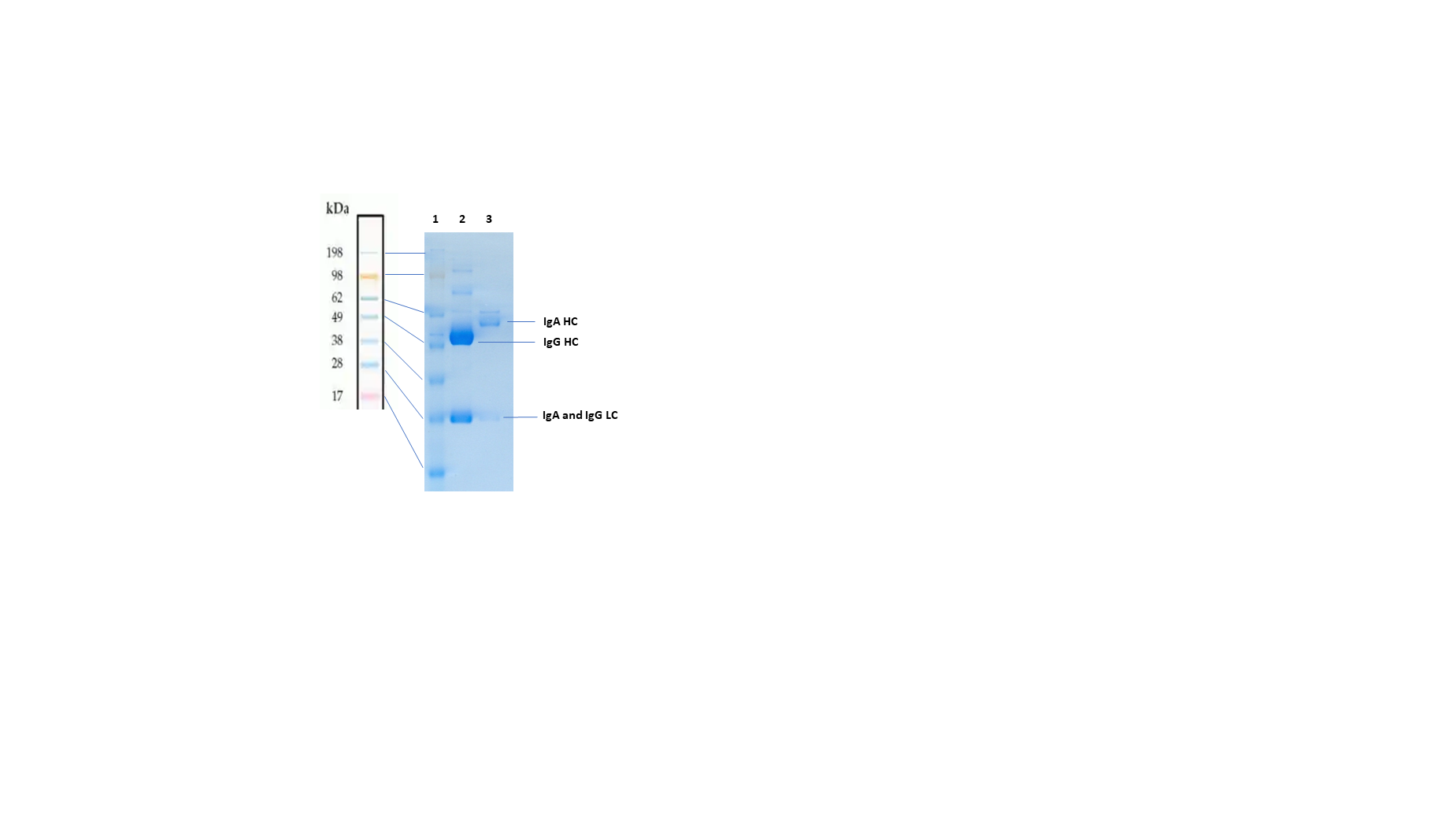

Supplement: Supplemental Material [file TEMI_A_2401940_SM3954.tiff]
